# Editorial: Rhizospheric microbiota-plant interactions: A bioremediation strategy for inorganic pollutants

**DOI:** 10.3389/fmicb.2023.1174634

**Published:** 2023-03-22

**Authors:** Khalid Oufdou, Anas Raklami, Eloísa Pajuelo, Adnane Bargaz, Mohammad Saghir Khan, Noureddine Bousserrhine, José A. Carrasco López

**Affiliations:** ^1^Laboratory of Microbial Biotechnologies, Agrosciences, and Environment (BioMAgE), Labeled Research Unit-CNRST N°4, Faculty of Sciences Semlalia, University Cadi Ayyad, Marrakech, Morocco; ^2^Agrobiosciences Program, College for Sustainable Agriculture and Environmental Sciences, University Mohammed VI Polytechnic (UM6P), Ben Guerir, Morocco; ^3^Department of Microbiology and Parasitology, Faculty of Pharmacy, University of Seville, Seville, Spain; ^4^Department of Agricultural Microbiology, Faculty of Agricultural Sciences, Aligarh Muslim University Aligarh, Aligarh, Uttar Pradesh, India; ^5^LEESU, Université Paris-Est Creteil, Ecole des Ponts, Creteil, France

**Keywords:** microbiome, phytoremediation, PGPR, metallo-tolerant bacteria, metal accumulator plants, wastes added-value

Environmental pollution with heavy metals has been accentuated by anthropogenic activities and constitute a major cause of ecosystem degradation and public health concern. Environmental reclamation assisted by plants and associated microbial communities is gaining momentum as an alternative to traditional physicochemical measures. This bioremediation technique offers several environmental benefits, notably improvement of soil fertility, promotion of ecosystem's biological diversity, and maintaining of aesthetics and health of landscapes. However, bioaccumulation of toxic elements in plant tissues can alter plant metabolism, destructs growth and yield, generates oxidative stress, and causes damage to DNA and membranes, etc.

Beneficial rhizobacteria, known as plant growth promoting bacteria (PGPR), establish a profitable relationship with plants and promote their growth under wide stress conditions. The mechanisms by which PGPR support plant growth can be categorized as direct (including nutrient acquisition such as phosphate solubilization, potassium and iron acquisition, and growth stimulation through phytohormones) and indirect (such as diminution of plant stress based on ACC deaminase activity and induction of systemic resistance). Besides, PGPR encompass species exhibiting biocontrol activities through the secretion of siderophores, antimicrobials, the inhibition of communication signals in the rhizosphere, etc. The ability of PGPR to colonize plant roots and form a robust and well-established biofilm also determines the effectiveness of microbial inoculants.

In this regard, it is known that metallo-tolerant PGPR assist the establishment and development of plants in metal-polluted environments. In addition, they can still promote plant growth and alleviate metal stress, which enhances the phytoremediation capacity of plants. Bacteria have evolved multiple resistance mechanisms to counteract the metal toxicity, including oxidation/reduction, extrusion from the cell through specific efflux pumps, complexation, precipitation, volatilization, etc., in order to survive in the harsh environments.

For this approach to be useful, metal-tolerant bacterial strains well adapted to particular regions must be employed. In this context, the study of phytomicrobiomes associated with plants is one of the most relevant areas of research. Omics technologies open new and exciting possibilities in the application of microbe-assisted phytoremediation by identifying, not only new bacterial strains with metal resistance and PGP traits, but also by providing comprehensive data concerning their effect on plants. This Research Topic is a compilation of updated research that explains the role of PGPR in the reclamation of metal-polluted soils.

Abid et al. examined the synergistic utilization of a hyperaccumulator plant, *Sedum alfredii*, and the cadmium-resistant *Bacillus* sp. M6, for Cd phytoremediation in soils. The bacterium improved the Cd phytoextraction capacity. Moreover, the addition of some amendments such as biochar, rhamnolipids, and glutamic acid further improved Cd mobilization and extraction. On the other side, the rhizosphere's metagenomics analysis showed changes in the rhizospheric bacterial populations upon *Bacillus* inoculation particularly in the relative abundance of ammonia-oxidizing prokaryotes and the *cadA* gene responsible for Cd extrusion from bacterial cells.

The work by Zhang et al. deals with Cd phytoremediation, using a different Cd hyperaccumulator, *Solanum nigrum*. The authors showed that inoculation with the Cd-tolerant strain *Bacillus* sp. PGP15, which displayed several plant growth promoting abilities, ameliorated plant growth and alleviated Cd stress. Moreover, full genome sequencing identified some transposable elements putatively involved in the evolution of heavy metal resistance in this bacterium.

Mghazli et al. investigated the revegetation of phosphate mine wastes particularly in semi-arid regions. This study focused on the isolation, characterization, and identification of indigenous bacterial strains from Moroccan phosphate mining wastes. After testing the PGP activities of the whole collection, the authors selected the best-performing bacteria *Stenotrophomonas rhizophila* which was able to improve plant growth and chlorophyll content. In addition, this study is the first to determine the PGP activity of *Brevibacterium anseongense*.

Finally, Sun et al. conducted a bibliometric review of scientific literature concerning rhizosphere microorganisms uncovering data from the last decade (2012–2022). The results have shown that the interest in this area is increasing which is evident from the rapidly increasing number of publications in many countries. Furthermore, cooperation networks between them are revealed, indicating high collaboration at the global level in this Research Topic.

In conclusion, we would like to emphasize that research on PGPR is very advanced at the academic level, and yet, the acceptance and actual field application of biofertilizers are still far from complete realization. Furthermore, differences can be observed in the application of PGPR amongst countries, both for crop optimization (agricultural) and environmental management. Estimation of the future market for biofertilizers depicts a promising scenario but requires the attention of the global scientific community and agro-biotechnology companies to fill the gaps between research and real practitioners. In this context, it is important to design useful and region-specific inoculants that could adapt well to particular stressful environmental conditions in order to develop what are called “tailored phytoremediation projects.” Furthermore, the development of local biotechnology companies for the introduction and popularization of this technology among farming communities in the near future could be a source of qualified jobs, generate opportunities, and foster social and scientific development. In addition, recently the use of several agricultural and/or industrial wastes as amendments, together with PGPR, is being proposed to improve and make phytoremediation profitable. This approach is important in the current scenario of the circular economy.

## Author contributions

All authors listed have made a substantial, direct, and intellectual contribution to the work and approved it for publication.

